# Effect of perceived workplace health support on absenteeism and presenteeism among Japanese workers: a prospective cohort study

**DOI:** 10.1093/joccuh/uiaf018

**Published:** 2025-03-17

**Authors:** Kazushirou Kurogi, Kazunori Ikegami, Hajime Ando, Akira Ogami

**Affiliations:** Department of Work Systems and Health, Institute of Industrial Ecological Sciences, University of Occupational and Environmental Health, Kitakyushu, Japan; Department of Health Care Center, Panasonic Health Insurance Organization, Osaka, Japan; Department of Work Systems and Health, Institute of Industrial Ecological Sciences, University of Occupational and Environmental Health, Kitakyushu, Japan; Sakurajyuji Fukuoka Hospital, Fukuoka, Japan; Department of Work Systems and Health, Institute of Industrial Ecological Sciences, University of Occupational and Environmental Health, Kitakyushu, Japan; Department of Work Systems and Health, Institute of Industrial Ecological Sciences, University of Occupational and Environmental Health, Kitakyushu, Japan

**Keywords:** perceived workplace health support, healthy and productive management, Japanese workers, presenteeism, absenteeism, occupational health

## Abstract

**Objectives:**

This study aimed to investigate the impact of perceived workplace health support (PWHS) on absenteeism and presenteeism among Japanese workers to determine the significance of health and productivity management in improving labor productivity.

**Methods:**

This prospective cohort study, using data from the Work Systems & Health Internet Research (WSHIR) study, involved 1879 Japanese workers aged 20-69 years. The intensity of PWHS was assessed using self-administered questionnaires. The participants were followed up from October 2021 to October 2022 to evaluate the incidence of absenteeism and presenteeism based on the level of PWHS.

**Results:**

The study findings revealed that higher PWHS significantly correlated with lower presenteeism, indicating better work productivity. Conversely, the relationship between PWHS and absenteeism was less clear, with no consistent trend observed across different levels of PWHS intensity.

**Conclusions:**

Enhanced PWHS was associated with reduced presenteeism among Japanese workers, underscoring the importance of workplace health support for improving employee productivity. This finding emphasizes the need for companies to focus on health promotion activities and recognize the potential of PWHS as a performance indicator in corporate health management.

## Teaser

Key points

What is already known on this topic:

- Amid concerns that Japan’s declining population and aging workforce will impact labor productivity, companies are increasingly focusing on maintaining and enhancing workers’ health.

What this study adds:

- Evaluating these health promotion efforts is crucial in terms of workers’ perceptions of workplace health support. This cohort study of the effects of perceived workplace health support (PWHS) on presenteeism and absenteeism found that higher PWHS among Japanese workers was associated with lower presenteeism and reduced health-related cost losses.

How this study might affect research, practice, or policy:

- PWHS has the potential to be a performance indicator for corporate health promotion and health management activities.

## 1. Introduction

Japan faces various challenges, such as a declining population and an aging society, which affect labor force availability and productivity.^1^ Companies are implementing work-style reforms and extending employment duration to maintain labor productivity.^2^ As part of these efforts, many companies focus on employee health management and active work to maintain and enhance workers’ health. In particular, the strategic approach to health management from a corporate perspective, known as healthy and productive management (H&PM), interests many Japanese companies.^3^ By investing in employee health and welfare and promoting H&PM, companies can positively affect employee engagement and organizational vitality, improve work productivity, and enhance corporate profits and performance.^4^ Clarifying the relationship between worker health support and labor productivity is crucial for determining the significance of H&PM.

Absenteeism (the state in which work opportunities are lost due to sick leave) and presenteeism (attending work while suffering from a disease or symptoms resulting in decreased work performance or productivity) are well-known indicators for evaluating labor productivity.^5^ Due to a reduced labor force, absenteeism leads to direct economic losses for companies. Presenteeism causes economic losses by worsening the illnesses of workers, hindering stable employment, and reducing labor productivity. The economic losses to companies due to these worker health issues are referred to as health-related costs, and it has been reported that health-related costs due to presenteeism account for a larger proportion than direct costs such as medical and drug costs and the cost of lost work opportunities due to absenteeism.^6^^,^^7^ To prevent absenteeism and presenteeism, companies must actively and proactively involve themselves in maintaining and promoting workers’ health.^4^^,^^8^

To maintain and promote health in the workplace, it is important not to leave health management solely to the workers themselves but to actively support health in the workplace. It is necessary to understand how employees perceive such support. Perceived organizational support (POS) represents workers’ evaluations and perceptions of their organization and has been defined as “the overall belief of workers regarding how much the organization values their opinions and cares about their well-being.”^9^^,^^10^ POS includes concepts beyond health, such as welfare benefits, and it is important to assess workers’ perceived workplace health support (PWHS) from this perspective. PWHS, which can be measured by the POS of workers toward ensuring a healthy lifestyle and encouraging participation in health promotion activities, has been shown to correlate positively with workers’ physical and mental health, reduction of anxiety and depressive symptoms, and improvement in work productivity.^11^^-^^14^

Although there have been some reports on the relationship between PWHS and presenteeism in cross-sectional studies, longitudinal studies evaluating the effects of PWHS on presenteeism and absenteeism are lacking. We conducted a prospective cohort study of occupational health using data from the Work Systems & Health Internet Research (WSHIR) study from October 2021 (baseline survey) to October 2022 (follow-up).^15^ Our study aimed to longitudinally evaluate data from the WSHIR study and examine how PWHS intensity affected absenteeism and presenteeism 1 year later.

## 2. Methods

### 2.1. Study design and setting

The WSHIR study was a prospective cohort study targeting workers and conducted by a research group at the University of Occupational and Environmental Health, Japan. The WSHIR study used an online research system provided by Cross Marketing Inc. (Tokyo, Japan) involving self-administered questionnaires. To enhance the validity of the WSHIR study, the authors adhered to the Checklist for Reporting Results of Internet E-Surveys.^11^^,^^16^ The details of the WSHIR study protocol have been published elsewhere.^15^

This cohort study used baseline and 1-year follow-up data from the WSHIR study. The baseline survey was conducted in October 2021, and the follow-up survey was conducted in October 2022. This study was conducted during the COVID-19 pandemic,^17^ which may have influenced both absenteeism and presenteeism due to varying infection rates and increased work-from-home practices. However, the study’s aim was to evaluate overall absenteeism and presenteeism patterns, including those potentially influenced by infectious diseases, and not to isolate the specific effects of COVID-19.

### 2.2. Participants

During the baseline survey, the participants were workers aged 20-69 years in Japan. Approximately 59 000 registered monitors were invited via email to participate, of whom 7300 responded and 5111 met the eligibility criteria for our study. After the questionnaires were submitted, checks for consistency and completeness of the survey responses were conducted. Using the 3 algorithms designed in this study, we detected fraudulent respondents.^15^ A total of 571 participants were excluded because of fraudulent responses, leaving 4540 eligible participants at baseline.

**Figure 1:**
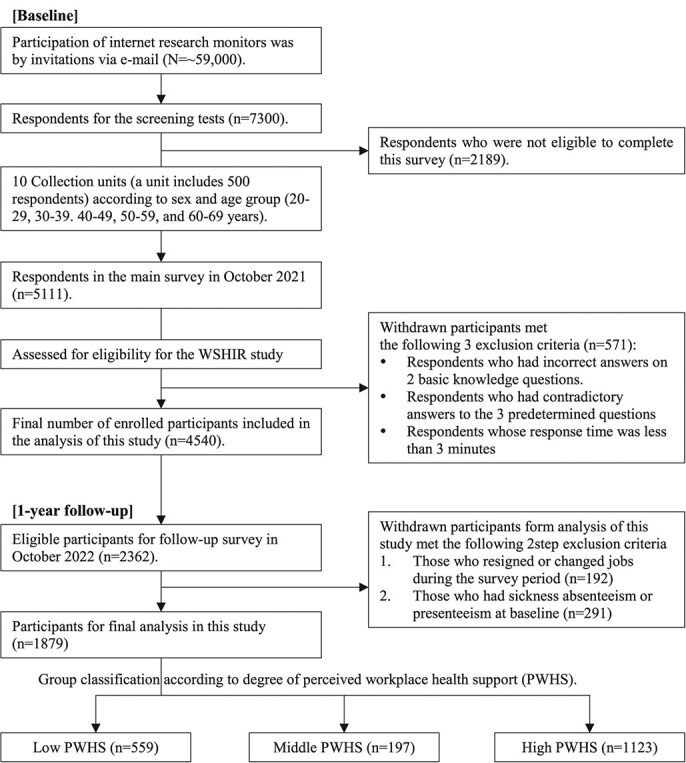
Flowchart of the study population selection.

For the 1-year follow-up survey, all 4540 participants were invited to participate through the internet survey system, with 2362 responses obtained (response rate: 52.0%). After excluding 192 individuals who reported retirement or job changes during the survey period and 291 participants who had absenteeism or presenteeism at baseline, the final analysis included 1879 participants (follow-up rate: 41.4%). A detailed flowchart of the study is shown in Figure 1.

This study was approved by the Ethics Committee of Medical Research of the University of Occupational and Environmental Health, Japan (R3-037). Written informed consent was obtained from the 1879 participants through the website. All procedures were followed in accordance with the Helsinki Declaration (1964, amended most recently in 2013).

### 2.3. Evaluation of PWHS

We evaluated PWHS intensity using the following questions: (1) Does your company help employees lead healthy lives and work? and (2) Does your company support employee participation in health-promoting activities? The participants were asked these questions using a 4-point Likert scale, with responses ranging from strongly agree to agree, disagree, and strongly disagree. In the present sample, the Cronbach α coefficient was .925.

PWHS intensity was calculated and evaluated by the scores (total score range: 0-2) obtained from the 2 questions, with 1 point given for “strongly agree” or “agree,” and 0 points for “strongly disagree” or “disagree.” A total score of 0 was categorized as low PWHS (*n* = 559), 1 as middle PWHS (*n* = 197), and 2 as high PWHS (*n* = 1123). This approach was adapted from a similar methodology used in our previous research.^11^ In other previous research examining the relationship between organizational health promotion activities and health-related quality of life, a comparable framework for assessing PWHS was also used, demonstrating the relevance of this approach.^12^ Therefore, our classification of PWHS was based on validated methods from prior studies, ensuring consistency and relevance in evaluating PWHS. The participants were categorized into these 3 groups based on PWHS intensity (Figure 1).

### 2.4. Assessment of absenteeism

In the follow-up survey, absenteeism was assessed by asking, “Over the past year, how many days have you been absent from work due to your own health problems or illness?” In Japan, the Labor Standards Law mandates the provision of annual paid leave to workers to meet certain requirements.^18^ The question used in this study did not differentiate between annual paid leave and sick leave specifically taken for health-related reasons; instead, our focus was on capturing overall health-related absenteeism. This approach aimed to identify workers who frequently took time off due to health issues, regardless of the type of leave used. Although there are no direct data on the average number of days of absenteeism due to illness in Japan, according to the Comprehensive Survey of Working Conditions conducted by the Ministry of Health, Labor, and Welfare in 2023, the average number of days of annual paid leave granted to workers by companies in 2022 was 17.6 days. The actual average number of days taken by workers was 10.9 days.^19^ In this study, we defined sickness absenteeism as the frequent use of 11 days or more of leave due to health reasons. The decision to set the threshold at 11 days was not based on the average values of paid leave but was instead intended to distinguish workers with a notably high absenteeism rate due to health problems. Individuals taking 11 or more days off for health reasons would be expected to experience significant health issues affecting their attendance, indicating high health-related absenteeism. Therefore, the research team’s consensus determined this cutoff to reflect frequent sickness absenteeism in the context of this study.

### 2.5. Assessment of presenteeism

Presenteeism was evaluated at the follow-up using the Quantity and Quality Method to measure work productivity.^20^ This method involved asking participants to compare the quantity and quality of their work during the most recent week with their usual level of performance. The responses were scored on an 11-point scale ranging from 0 (unable to work at all) to 10 (normal). Presenteeism loss, an index of labor productivity evaluation, was calculated using the scores obtained for both the quantity and quality of work according to the following formula, where a higher presenteeism loss was considered indicative of poorer labor productivity metrics:

Presenteeism loss = 1 – Quantity (range: 0-10) × Quality (range: 0-10)/100.

Based on prior research,^21^ the cutoff value for presenteeism loss was defined as scores in the top 10% (above 0.5), indicating the presence of presenteeism. This study used the top 10% of presenteeism scores as the threshold to define high presenteeism. Since no established cutoff point exists for presenteeism, this classification was based on previous studies and the research team’s consensus for identifying significant productivity loss.

### 2.6. Outcome and covariates

The outcomes of interest at follow-up were sickness absenteeism and presenteeism with PWHS intensity as the exposure variable (high PWHS: reference). Confounders considered included personal characteristics (gender; age groups: 20-29, 30-39, 40-49, 50-59, 60-69 years; educational background: middle/high school, junior college/vocational school, university/graduate school; marital status) and work-related factors (job type: regular employees, managers, others; standard industrial classification: primary and secondary industry, tertiary industry; workplace size: ≤49, 50-199, 200-999, ≥1000 employees, unclear; average working hours per week: ≤29, 30-39, 40-49, 50-59, ≥60 hours; and income status: ≤2.99 million yen, 3.00-4.99 million yen, 5.00-9.99 million yen, ≥10.00 million yen, decline to answer).

### 2.7. Statistical analysis

The analysis results of 1879 participants were used to evaluate the incidence of sickness absenteeism and presenteeism 1 year later according to PWHS intensity at baseline. The incidence of sickness absenteeism and presenteeism across PWHS groups was compared using chi-squared tests. To assess the incidence of absenteeism and presenteeism according to PWHS intensity, we employed a logistic regression model and a generalized linear model with a binomial response. Two models were analyzed: one adjusted for gender and age, the other a multivariate model adjusted for gender, age, education, marital status, job type, standard industrial classification, workplace size, average working hours per week, and income status. The significance threshold was set at *P* < .05. All statistical analyses were conducted using IBM SPSS software (version 23.0).

## 3. Results

Table 1 presents the basic information of the participants in this study. In terms of gender distribution, there were 965 males (51.4%) and 914 females (48.6%). A lower participation rate was observed among the younger age group in their 20s. When examining PWHS intensity, a higher proportion of participants aged 60-69 years fell into the high PWHS group. Regarding educational background, those with middle- or high-school education were predominantly in the low PWHS group. In contrast, those with university or graduate degrees were more likely to be in the high PWHS group. In the standard industrial classification, no participants were classified as working in the primary industries, suggesting that these industries may lack formal employment relationships and systematic labor management. Employees working in companies with more than 1000 workers comprised a higher proportion of the high PWHS group. Those working over 50 hours per week and with incomes of less than 3 million yen were likelier to be in the low PWHS group. Table 1 presents the demographic characteristics of the participants at baseline and follow-up. As noted, younger age groups had a lower response rate in the follow-up survey, resulting in a smaller sample size for this subgroup. These differences are attributable to participant dropout rather than systematic sampling bias. Therefore, no statistical tests were performed to compare baseline characteristics, as the purpose of this table is to describe the overall demographic distribution rather than establish statistical differences between groups, in line with the STROBE (Strengthening the Reporting of Observational Studies in Epidemiology) guidelines.^22^^,^^23^

**Table 1:** Participants’ characteristics.

	**PWHS_groups** ^**a**^					
**Items**	**Low**		**Middle**		**High**	
	**(*n* = 559)**		**(*n* = 197)**		**(*n* = 1123)**	
Gender, male, *n* (%)	307	(54.9)	93	(47.2)	565	(50.3)
Age, *n* (%), y						
20-29	44	(7.9)	23	(11.7)	138	(12.3)
30-39	117	(20.9)	38	(19.3)	185	(16.5)
40-49	159	(28.4)	41	(20.8)	242	(21.5)
50-59	144	(25.8)	48	(24.4)	254	(22.6)
60-69	95	(17.0)	47	(23.9)	304	(27.1)
Educational background, *n* (%)						
Middle-school/high-school	150	(26.8)	43	(21.8)	207	(18.4)
Junior college/vocational school	111	(19.9)	38	(19.3)	243	(21.6)
University/graduate school	298	(53.3)	116	(58.9)	673	(59.9)
Marital status, unmarried, *n* (%)	293	(52.4)	98	(49.7)	470	(41.9)
Job type, *n* (%)						
Regular employees	363	(64.9)	113	(57.4)	573	(51.0)
Managers	45	(8.1)	23	(11.7)	113	(10.1)
Others	151	(27.0)	61	(31.0)	437	(38.9)
Standard industrial classification, *n* (%)						
Primary industry	0	(0.0)	0	(0.0)	0	(0.0)
Secondary industry	135	(24.2)	48	(24.4)	287	(25.6)
Tertiary industry	424	(75.8)	149	(75.6)	836	(74.4)
Workplace size (no. of employees), *n* (%)						
≤49	247	(44.2)	81	(41.1)	452	(40.2)
50-199	113	(20.2)	46	(23.4)	190	(16.9)
200-999	90	(16.1)	29	(14.7)	185	(16.5)
≥1000	85	(15.2)	34	(17.3)	267	(23.8)
Unclear	24	(4.3)	7	(3.6)	29	(2.6)
Average working hours per week, *n* (%)						
≤29	55	(9.8)	25	(12.7)	128	(11.4)
30-39	87	(15.6)	43	(21.8)	240	(21.4)
40-49	279	(49.9)	92	(46.7)	580	(51.6)
50-59	87	(15.6)	21	(10.7)	121	(10.8)
≥60	51	(9.1)	16	(8.1)	54	(4.8)
Income status, *n* (%), million yen						
≤2.99	131	(23.4)	39	(19.8)	215	(19.1)
3.00-4.99	174	(31.1)	75	(38.1)	341	(30.4)
5.00-6.99	87	(15.6)	34	(17.3)	191	(17.0)
7.00-9.99	61	(10.9)	20	(10.2)	135	(12.0)
≥10.00	15	(2.7)	9	(4.6)	87	(7.7)
Declined to answer	91	(16.3)	20	(10.2)	154	(13.7)

Abbreviation: PWHS, perceived workplace health support.

a PWHS groups according to the intensity of perceived workplace health support.

Table 2 shows the incidences of sickness absenteeism and presenteeism. The most common response to sickness absenteeism over the year was zero days, followed by less than 10 days. A total of 86 participants (4.6%) had notable sickness absenteeism (absent for 11 days or more). Regarding PWHS intensity, the middle PWHS group had a higher percentage of sickness absenteeism (10 participants [5.1%]), with the low and high PWHS groups showing similar rates (*P* = .937). The mean (SD) of presenteeism loss related to presenteeism was 0.07 (0.20) points overall, with 142 participants (7.6%) experiencing presenteeism. The low PWHS group had a higher presenteeism loss (0.09 [0.23] points) and a higher incidence rate of presenteeism (58 participants [10.4%]). Chi-squared tests indicated significant differences in the incidence of presenteeism across the PWHS groups (*P* = .010). The higher the PWHS, the lower the mean score of presenteeism loss (indicating better work productivity) and the lower the incidence of presenteeism.

**Table 2:** Incidences of sickness absenteeism and presenteeism.

			**PWHS_groups** ^**a**^						
**Items**	**Total (n = 1879)**		**Low**		**Middle**		**High**		***P* values**
			**(*n* = 559)**		**(*n* = 197)**		**(*n* = 1123)**		
Absenteeism									
Distribution of sick leave days for workers (d/y), *n* (%)									
0	1204 (64.1)		367	(65.7)	111	(56.3)	726	(64.6)	
1-5	483 (25.7)		146	(26.1)	55	(27.9)	282	(25.1)	
6-10	106 (5.6)		21	(3.8)	21	(10.7)	64	(5.7)	
11-15	41 (2.2)		14	(2.5)	8	(4.1)	19	(1.7)	
16-30	24 (1.3)		6	(1.1)	2	(1.0)	16	(1.4)	
≥31	21 (1.1)		5	(0.9)	0	(0.0)	16	(1.4)	
Workers with a high absenteeism ratio, *n* (%)	86 (4.6)		25	(4.5)	10	(5.1)	51	(4.5)	.937^b^
Presenteeism									
Scores using the Quantity and Quality (QQ) method, mean (SD)	0.07 (0.20)		0.09	(0.23)	0.07	(0.21)	0.05	(0.18)	
Workers with notable productivity losses, *n* (%)	142 (7.6)		58	(10.4)	14	(7.1)	70	(6.2)	.010^b^

a Groups categorized according to the intensity of perceived workplace health support (PWHS).

b Chi-squared test results.

Table 3 presents the odds ratios (ORs) and 95% CIs for the rates of sickness absenteeism and presenteeism associated with PWHS intensity across different models. In the gender-age–adjusted model, the occurrence rate of sickness absenteeism was not significantly different between the PWHS groups. This trend was also observed in the multivariate-adjusted model, which included adjustments for other personal characteristics and work-related factors. The occurrence rate of presenteeism decreased with increasing PWHS intensity in both models. Notably, the high PWHS group had a significantly lower presenteeism occurrence rate than the low PWHS group in the gender-age model (OR = 1.65; 95% CI, 1.14-2.39; *P* = .007). A similar trend was observed in the multivariate-adjusted model, which also adjusted for personal characteristics and work-related factors (OR = 1.60; 95% CI, 1.09-2.35; *P* = .017).

**Table 3:** Association between the rates of sickness absenteeism and presenteeism among groups according to perceived workplace health support (PWHS).

Outcomes PWHS_group^a^	**Gender-age adjusted**			**Multivariate** ^**b**^		
	**OR**	**(95% CI)**	***P***	**OR**	**(95% CI)**	***P***
Absenteeism			.882^c^			.637^c^
Low PWHS	1.03	(0.63-1.69)	.911	1.11	(0.66-1.86)	.689
Middle PWHS	1.12	(0.56-2.25)	.747	1.28	(0.63-2.61)	.496
High PWHS	Ref.			Ref.		
Presenteeism			.008^c^			.018^c^
Low PWHS	1.65	(1.14-2.39)	.007	1.60	(1.09-2.35)	.017
Middle PWHS	1.15	(0.63-2.09)	.644	1.16	(0.63-2.12)	.629
High PWHS	Ref.			Ref.		

Abbreviation: OR, odds ratio.

a PWHS groups according to the intensity of perceived workplace health support (PWHS).

b Multivariate: adjusted for sex, age, education, marital status, job types, standard industrial classification, workplace size, average working hours per week, and income status.

c *P* for trend.

## 4. Discussion

This longitudinal study investigated the impact of PWHS on presenteeism and absenteeism. Individuals with high PWHS were more likely to have reduced presenteeism 1 year later. Specifically, even after adjusting for personal characteristics and work-related factors, high PWHS was significantly associated with a lower incidence of presenteeism, compared with low PWHS. Ensuring a healthy lifestyle for workers in the workplace and providing health promotion activities can effectively reduce presenteeism.

Regarding the relationship between workplace health support and presenteeism, previous studies have reported that workplace wellness programs effectively improve employee presenteeism.^24^^,^^25^ Improvements in psychological stress have been observed through undergoing stress checks and improvements in the workplace environment.^26^ Cross-sectional studies of the relationship between PWHS and presenteeism have shown that higher PWHS is associated with an improved quality of life related to physical and mental health.^12^ Our longitudinal study supports previous findings and further emphasizes the importance of workplace support in reducing the incidence of presenteeism 1 year later.

In particular, a significant reduction in presenteeism was observed between high and low PWHS groups. Although this study did not consider personal health risks or lifestyle factors as adjustment factors, presenteeism was reduced by PWHS. Presenteeism, which impairs productivity due to chronic conditions such as headaches, back pain, mental illness, or allergy symptoms, is a significant economic burden for companies.^8^^,^^27^^,^^28^ To mitigate presenteeism, companies must take an interest in employee health support, and evaluating employees’ PWHS to promote health support can be considered highly effective.

In this study, the incidence rate of sickness absenteeism was higher in the middle PWHS group than in the high PWHS group but was similar between the low and high PWHS groups. However, these differences were not statistically significant. There was no dose–response relationship regarding the reduction in sickness absenteeism according to PWHS intensity, and no consistent trend was observed between PWHS and sickness absenteeism. Previous studies have reported that workplace health promotion activities do not reduce absenteeism.^14^^,^^29^ Reasons for working despite poor health include constraints against absenteeism (eg, strict absence policies, job insecurity), job demands, and negative interpersonal relations due to absenteeism.^30^^-^^32^ Increased job demands can lead to a shift from absenteeism to presenteeism.^33^ These findings suggest that absenteeism is not influenced by PWHS intensity and that distinctive perceptions of attendance in Japan may also play a role. A survey of Japanese workers during the early COVID-19 outbreak (May 2020) found that 62.2% of symptomatic individuals attended work within 7 days of onset, suggesting a societal trend to attend work despite poor health, which could have influenced the results.^34^ Nonabsenteeism combines those without illnesses requiring rest and those who cannot take time off despite having such illnesses, making it an uncertain metric as a productivity indicator.

Presenteeism accounts for a significant proportion of health-related costs. This study found a strong association between PWHS and presenteeism. Enhancing PWHS is likely to be effective in preventing health-related costs. Corporate health promotion efforts contribute to the reduction of presenteeism,^24^^,^^35^ and focusing on workers’ PWHS is essential in controlling presenteeism. Furthermore, PWHS could serve as a performance indicator in corporate health promotion and H&PM activities.

## 5. Limitations

This study had several limitations. First, it was conducted online, limiting participants to those with internet access, residing in Japan, and registered in the online survey system of Cross Marketing, Inc. Therefore, the survey sample may not represent the general workforce, restricting the generalizability of the results. To mitigate bias, we divided the participants according to gender and age. Second, the study did not inquire about the participants’ baseline lifestyle habits or health risks. Consequently, the group with low PWHS at baseline may have been significantly affected by poor lifestyle habits or health conditions, potentially influencing absenteeism and presenteeism. However, prior research (a protocol study) evaluated the participants’ mental health status, confirming that our study’s participants were comparable to the general population,^15^ suggesting a minimal impact from these factors. Third, the survey was conducted during the COVID-19 pandemic, which may have led to differing effects of PWHS on absenteeism and presenteeism compared with more stable periods. For instance, many companies implemented work-from-home policies or enhanced health management to prevent COVID-19.^36^ Nevertheless, this longitudinal survey analysis, after adjusting for confounding factors, is considered to provide an acceptable level of validity. Lastly, participants who changed jobs or retired during the follow-up period were excluded to maintain workplace exposure and outcome measurement consistency. Although this approach minimizes bias, it may also introduce a “Healthy Worker Effect,” as only employed workers were included. However, this is an inherent limitation in workplace studies and is unavoidable when focusing on active workers.

## 6. Conclusion

This study’s findings indicated that lower PWHS intensity was associated with a higher incidence rate of presenteeism 1 year later, indicating a potential effect of PWHS intensity on the occurrence of presenteeism. Encouraging healthy lifestyles and participation in health promotion activities by companies may reduce presenteeism. Therefore, PWHS could serve as a performance indicator in corporate health promotion and H&PM activities.

## Data Availability

All data produced in this study are available upon reasonable request from the authors.

## References

[1] Statistics Bureau of Japan. *Statistical Handbook of Japan*. 2024. Accessed January 20, 2025. https://www.stat.go.jp/english/data/handbook/index.html.

[2] The Japan Institute for Labour Policy and Training (JILPT) . Challenges facing Japan: work styles and labor shortages. MHLW's white paper on the labor economy 2019. Jpn Lab Issues. 2020;4(23):

[3] Ministry of Economy Trade and Industry. Announcement of organizations selected under the 2023 certified health & productivity management outstanding organizations recognition program. Accessed January 20, 2025. https://www.meti.go.jp/english/press/2023/0308_004.html

[4] Mori K, Nagata T, Nagata M, et al. Development, success factors, and challenges of government-led health and productivity management initiatives in Japan. J Occup Environ Med. 2021;63(1):18-26. 10.1097/JOM.0000000000002002 32826547 PMC7773166

[5] Gosselin E, Lemyre L, Corneil W. Presenteeism and absenteeism: differentiated understanding of related phenomena. J Occup Health Psychol. 2013;18(1):75-86. 10.1037/a0030932 23276197

[6] Loeppke R, Taitel M, Haufle V, Parry T, Kessler RC, Jinnett K. Health and productivity as a business strategy: a multiemployer study. J Occup Environ Med. 2009;51(4):411-428. 10.1097/JOM.0b013e3181a39180 19339899

[7] Nagata T, Mori K, Ohtani M, et al. Total health-related costs due to absenteeism, presenteeism, and medical and pharmaceutical expenses in Japanese employers. J Occup Environ Med. 2018;60(5):e273-e280. 10.1097/JOM.0000000000001291 29394196 PMC5959215

[8] Yano Y, Kanegae H, Node K, et al. The associations of the national health and productivity management program with corporate profits in Japan. Epidemiol Health. 2022;44:e2022080. 10.4178/epih.e2022080 36177978 PMC10106540

[9] Eisenberger R, Huntington R, Hutchison S, Sowa D. Perceived organizational support. J Appl Psychol. 1986;71(3):500-507. 10.1037/0021-9010.71.3.500

[10] Eisenberger R, Stinglhamber F. Perceived Organizational Support: Fostering Enthusiastic and Productive Employees. American Psychological Association; 2011.

[11] Ikegami K, Ando H, Kurogi K, Ogami A. Perceived workplace health support and severe psychological distress among Japanese workers: a prospective cohort study. J Occup Environ Med. 2023;65(12):992-997. 10.1097/JOM.0000000000002936 37505081

[12] Kurogi K, Ikegami K, Eguchi H, et al. A cross-sectional study on perceived workplace health support and health-related quality of life. J Occup Health. 2021;63(1):e12302. 10.1002/1348-9585.12302 34877733 PMC8652405

[13] Laing SS, Jones SMW. Anxiety and depression mediate the relationship between perceived workplace health support and presenteeism: a cross-sectional analysis. J Occup Environ Med. 2016;58(11):1144-1149. 10.1097/JOM.0000000000000880 27820765

[14] Chen L, Hannon PA, Laing SS, et al. Perceived workplace health support is associated with employee productivity. Am J Health Promot. 2015;29(3):139-146. 10.4278/ajhp.131216-QUAN-645 25559250

[15] Ikegami K, Yoshimoto Y, Baba H, Sekoguchi S, Ando H, Ogami A. Study protocol and preliminary results of the impact of occupational health workers' activities on their health: nationwide prospective internet-based survey. JMIR Form Res. 2022;6(7):e35290. 10.2196/35290 35900807 PMC9337616

[16] Eysenbach G . Improving the quality of web surveys: the checklist for reporting results of internet E-surveys (CHERRIES). J Med Internet Res. 2004;6(3):e34. 10.2196/jmir.6.3.e34 15471760 PMC1550605

[17] Karako K, Song P, Chen Y, Karako T. COVID-19 in Japan during 2020-2022: characteristics, responses, and implications for the health care system. J Glob Health. 2022;12:03073. 10.7189/jogh.12.03073 36227719 PMC9559364

[18] Labor Standards Act [Japanese/English]. Japanese Law Translation. Accessed January 20, 2025. https://www.japaneselawtranslation.go.jp/en/laws/view/3567

[19] Japanese workers took 62% of the paid leave allocation in 2022: government survey. Kyodo News; November 26, 2023. Accessed January 20, 2025. https://english.kyodonews.net/news/2023/11/b3593a159d27-japan-workers-took-62-of-paid-leave-allocation-in-2022-govt-survey.html#:~:text=Japan%20saw

[20] Brouwer WB, Koopmanschap MA, Rutten FF. Productivity losses without absence: measurement validation and empirical evidence. Health Policy. 1999;48(1):13-27. 10.1016/s0168-8510(99)00028-7 10539583

[21] Mori T, Nagata T, Nagata M, Otani M, Fujino Y, Mori K. The impact of diabetes status on presenteeism in Japan. J Occup Environ Med. 2020;62(8):654-661. 10.1097/JOM.0000000000001922 32472846

[22] von Elm E, Altman DG, Egger M, et al. The strengthening the reporting of observational studies in epidemiology (STROBE) statement: guidelines for reporting observational studies. Ann Intern Med. 2007;147(8):573-577. 10.7326/0003-4819-147-8-200710160-00010 17938396

[23] Vandenbroucke JP, von Elm E, Altman DG. Strengthening the reporting of observational studies in epidemiology (STROBE): explanation and elaboration. Ann Intern Med. 2007;147(8):163. 10.7326/0003-4819-147-8-200710160-00010-w1 17938389

[24] Michishita R, Jiang Y, Ariyoshi D, et al. The introduction of an active rest program by workplace units improved the workplace vigor and presenteeism among workers: a randomized controlled trial. J Occup Environ Med. 2017;59(12):1140-1147. 10.1097/JOM.0000000000001121 28816734

[25] Cancelliere C, Cassidy JD, Ammendolia C, Côté P. Are workplace health promotion programs effective at improving presenteeism in workers? A systematic review and best evidence synthesis of the literature. BMC Public Health. 2011;11(1):395. 10.1186/1471-2458-11-395 21615940 PMC3123596

[26] Imamura K, Asai Y, Watanabe K, et al. Effect of the National Stress Check Program on mental health among workers in Japan: a 1-year retrospective cohort study. J Occup Health. 2018;60(4):298-306. 10.1539/joh.2017-0314-OA 29669966 PMC6078839

[27] Goetzel RZ, Long SR, Ozminkowski RJ, Hawkins K, Wang S, Lynch W. Health, absence, disability, and presenteeism cost estimates of certain physical and mental health conditions affecting U.S. employers. J Occup Environ Med. 2004;46(4):398-412. 10.1097/01.jom.0000121151.40413.bd 15076658

[28] Allen D, Hines EW, Pazdernik V, Konecny LT, Breitenbach E. Four-year review of presenteeism data among employees of a large United States health care system: a retrospective prevalence study. Hum Resour Health. 2018;16(1):59. 10.1186/s12960-018-0321-9 30413168 PMC6234777

[29] Edmunds S, Stephenson D, Clow A. The effects of a physical activity intervention on employees in small and medium enterprises: a mixed methods study. Work. 2013;46(1):39-49. 10.3233/WOR-121523 23241703

[30] Johns G . Attendance dynamics at work: the antecedents and correlates of presenteeism, absenteeism, and productivity loss. J Occup Health Psychol. 2011;16(4):483-500. 10.1037/a0025153 21875212

[31] Miraglia M, Johns G. Going to work ill: a meta-analysis of the correlates of presenteeism and a dual-path model. J Occup Health Psychol. 2016;21(3):261-283. 10.1037/ocp0000015 26550958

[32] Suárez MJ, Muñiz C. Unobserved heterogeneity in work absence. Eur J Health Econ. 2018;19(8):1137-1148. 10.1007/s10198-018-0962-6 29468342

[33] Aronsson G, Hagberg J, Björklund C, et al. Health and motivation as mediators of the effects of job demands, job control, job support, and role conflicts at work and home on sickness presenteeism and absenteeism. Int Arch Occup Environ Health. 2021;94(3):409-418. 10.1007/s00420-020-01591-w 33099673 PMC8032575

[34] Machida M, Nakamura I, Saito R, et al. The actual implementation status of self-isolation among Japanese workers during the COVID-19 outbreak. Trop Med Health. 2020;48(1):63. 10.1186/s41182-020-00250-7 32765185 PMC7396451

[35] Ministry of Economy, Trade and Industry. METI shares the health and productivity management scores of 2,000 Japanese companies. Accessed January 20, 2025. https://www.meti.go.jp/english/mobile/2022/20220615001en.html

[36] Suzuki H, Miyamoto T, Hamada A, et al. A guide for businesses and employers responding to novel coronavirus disease (COVID-19): 4th edn. J Occup Health. 2021;63(1):e12225. 10.1002/1348-9585.12225 34713533 PMC8250361

